# Supplementary figures and images for: Structural and mechanistic insights into the MCM8/9 helicase complex
Source: eLife. 2023 Aug 3;12:RP87468. doi: 10.7554/eLife.87468 (PMC10400076; doi:10.7554/eLife.87468)

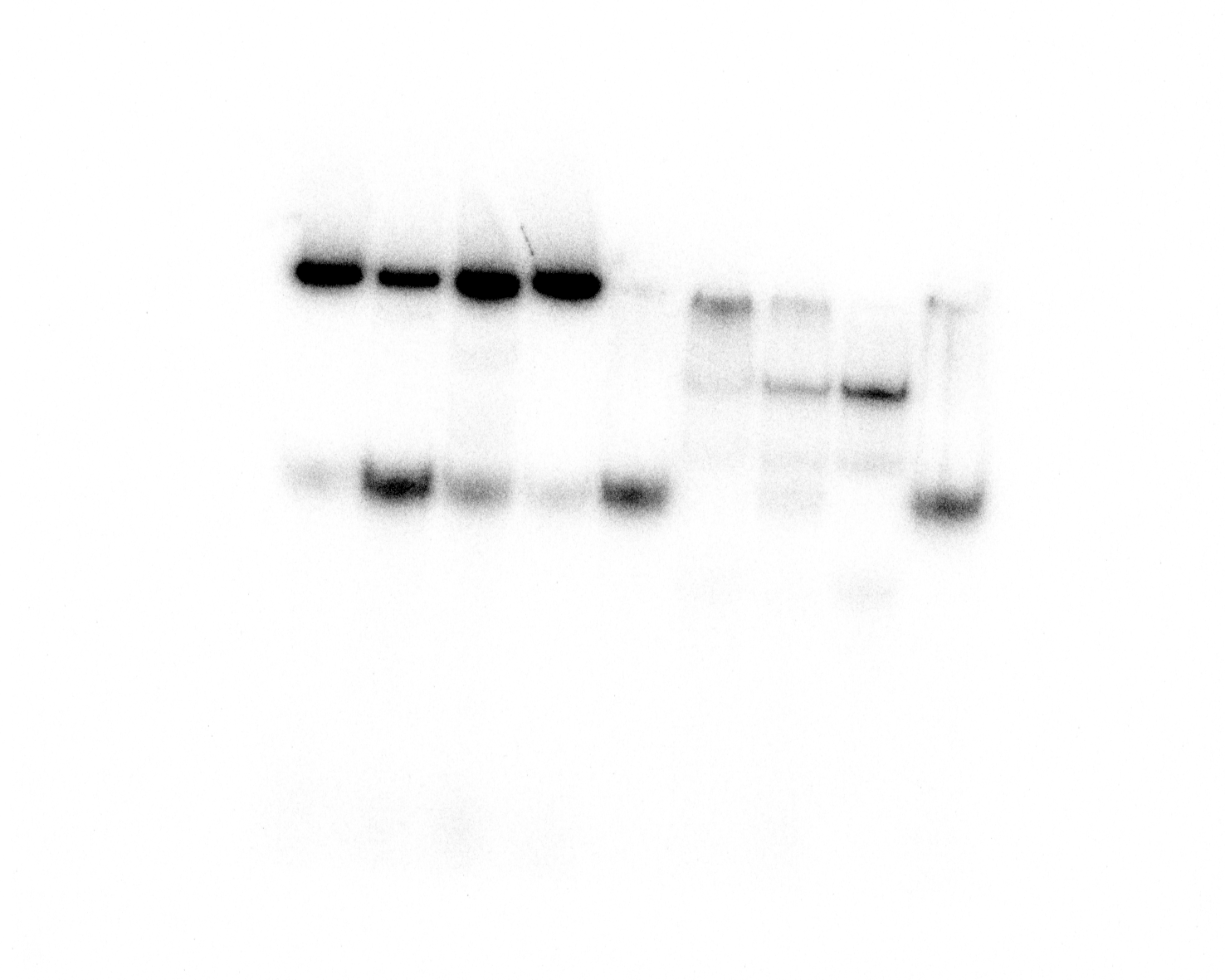

Supplement: Figure 4—source data 1. — Representative autoradiograph of the DNA unwinding reaction was conducted using a 32P-labeled ssDNA oligo annealed to the M13mp18 as DNA substrate in the presence of HROB-MBM or HROB-CTD with purified hMCM8/9. Control, DNA substrate without proteins. [file elife-87468-fig4-data1.zip › Figure 4 source data -1/Figure 4 source data -1-1.tif]

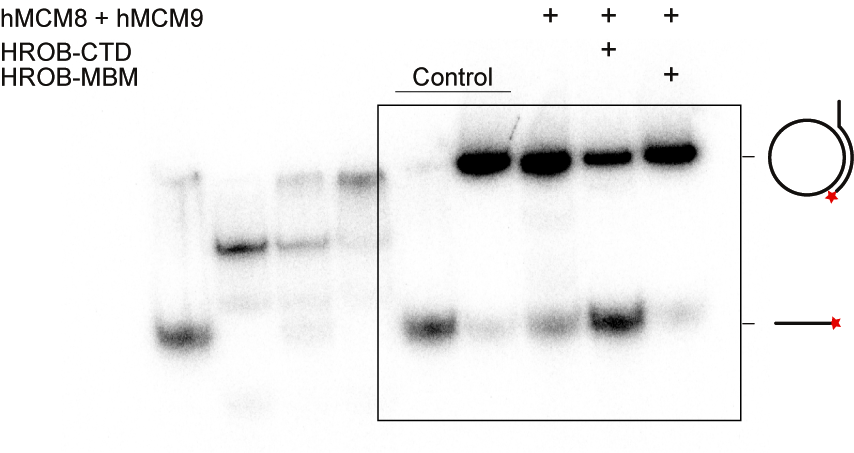

Supplement: Figure 4—source data 1. — Representative autoradiograph of the DNA unwinding reaction was conducted using a 32P-labeled ssDNA oligo annealed to the M13mp18 as DNA substrate in the presence of HROB-MBM or HROB-CTD with purified hMCM8/9. Control, DNA substrate without proteins. [file elife-87468-fig4-data1.zip › Figure 4 source data -1/FIGURE4-Source data 1.tif]

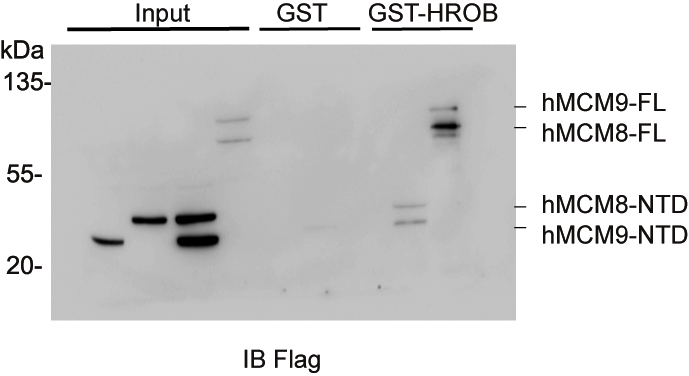

Supplement: Figure 4—source data 2. — Detection by western blot of MCM8/9-FL or MCM8/9-NTD co-precipitated by bead-bound GST or GST-HROB. [file elife-87468-fig4-data2.zip › Figure 4 source data -2/Figure 4 source data -2.tif]

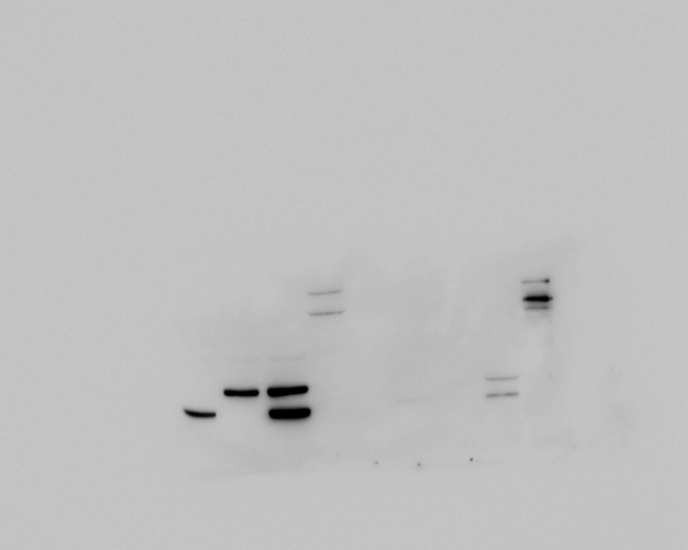

Supplement: Figure 4—source data 2. — Detection by western blot of MCM8/9-FL or MCM8/9-NTD co-precipitated by bead-bound GST or GST-HROB. [file elife-87468-fig4-data2.zip › Figure 4 source data -2/Figure 4 source data 2.tif]

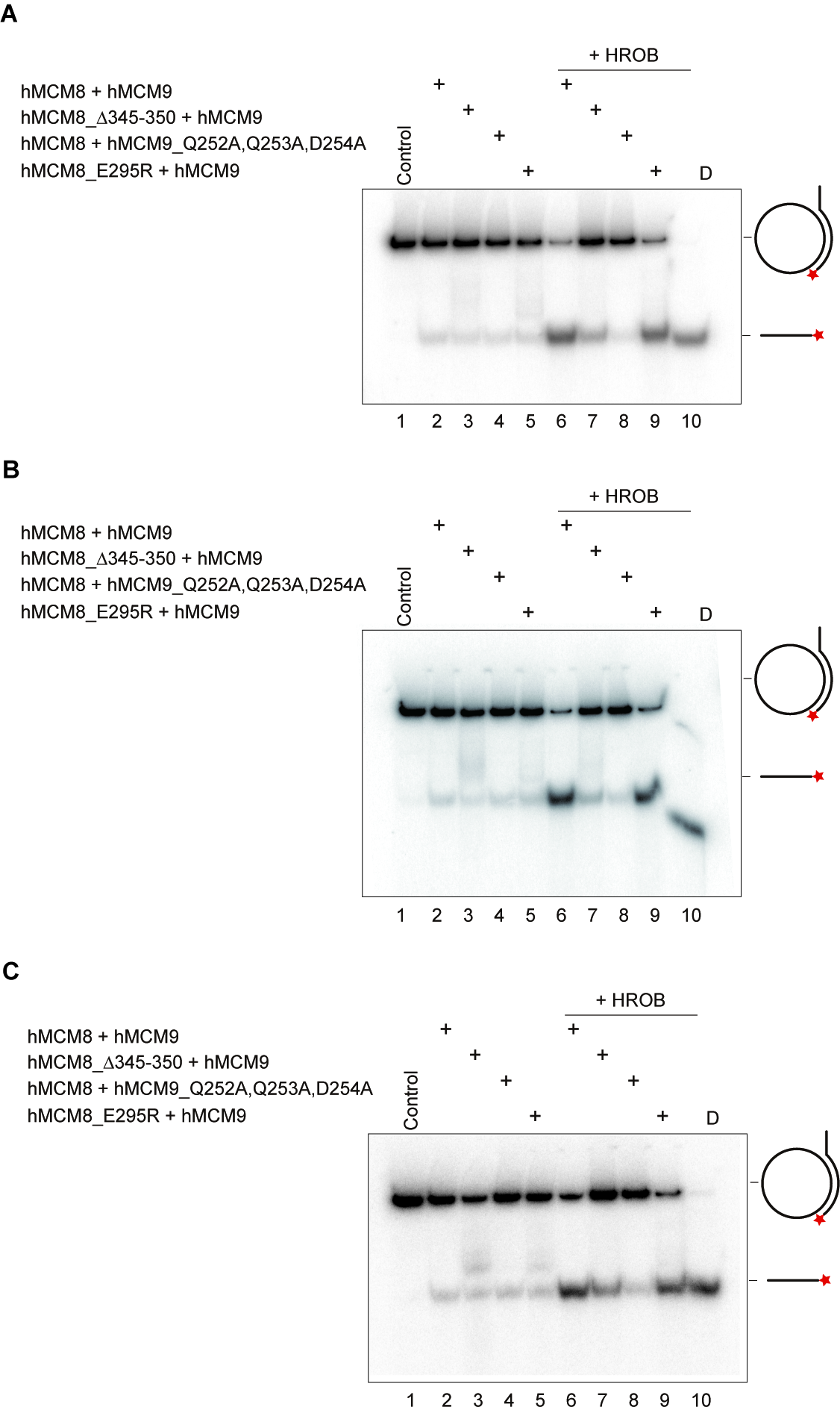

Supplement: Figure 5—source data 1. — Representative autoradiograph of the DNA unwinding reaction was conducted using a 32P-labeled ssDNA oligo annealed to the M13mp18 as DNA substrate in the presence of HROB with purified MCM8/9 or mutants as indicated. The assays were repeated at least three times. [file elife-87468-fig5-data1.zip › Figure 5 source data -1/FIGURE5-Source data 1.tif]

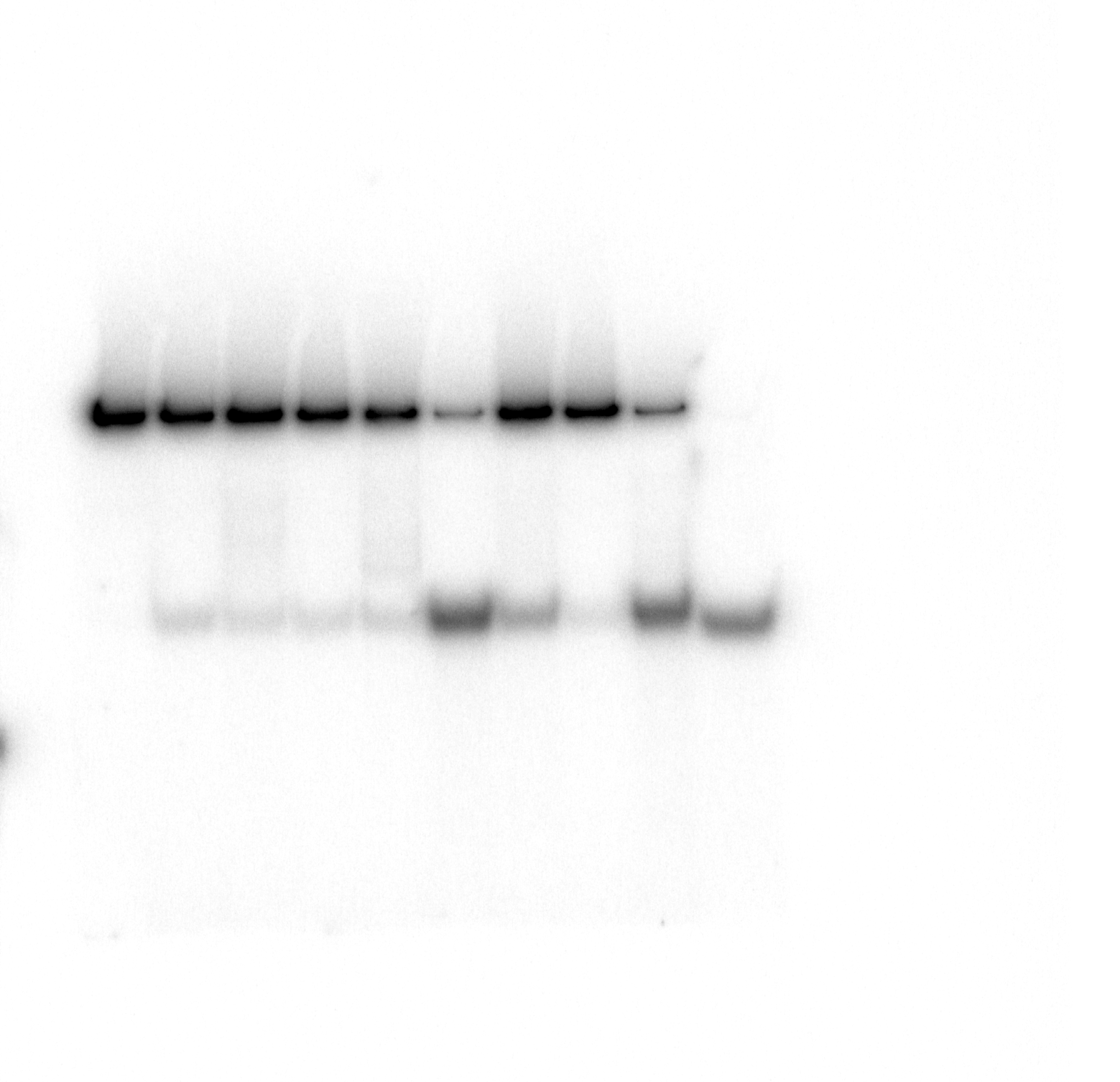

Supplement: Figure 5—source data 1. — Representative autoradiograph of the DNA unwinding reaction was conducted using a 32P-labeled ssDNA oligo annealed to the M13mp18 as DNA substrate in the presence of HROB with purified MCM8/9 or mutants as indicated. The assays were repeated at least three times. [file elife-87468-fig5-data1.zip › Figure 5 source data -1/source data 1-1.tif]

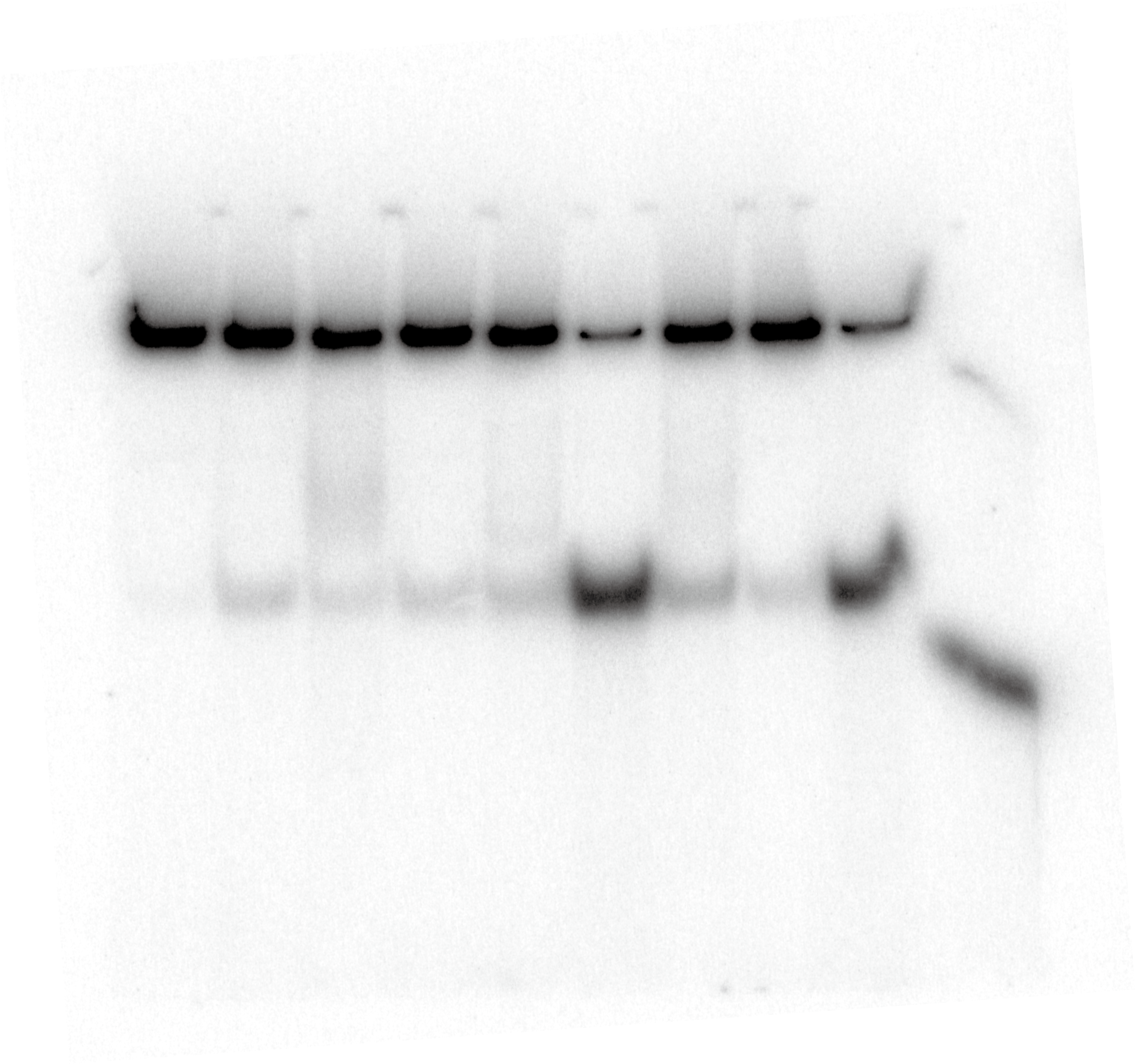

Supplement: Figure 5—source data 1. — Representative autoradiograph of the DNA unwinding reaction was conducted using a 32P-labeled ssDNA oligo annealed to the M13mp18 as DNA substrate in the presence of HROB with purified MCM8/9 or mutants as indicated. The assays were repeated at least three times. [file elife-87468-fig5-data1.zip › Figure 5 source data -1/source data 1-2.tif]

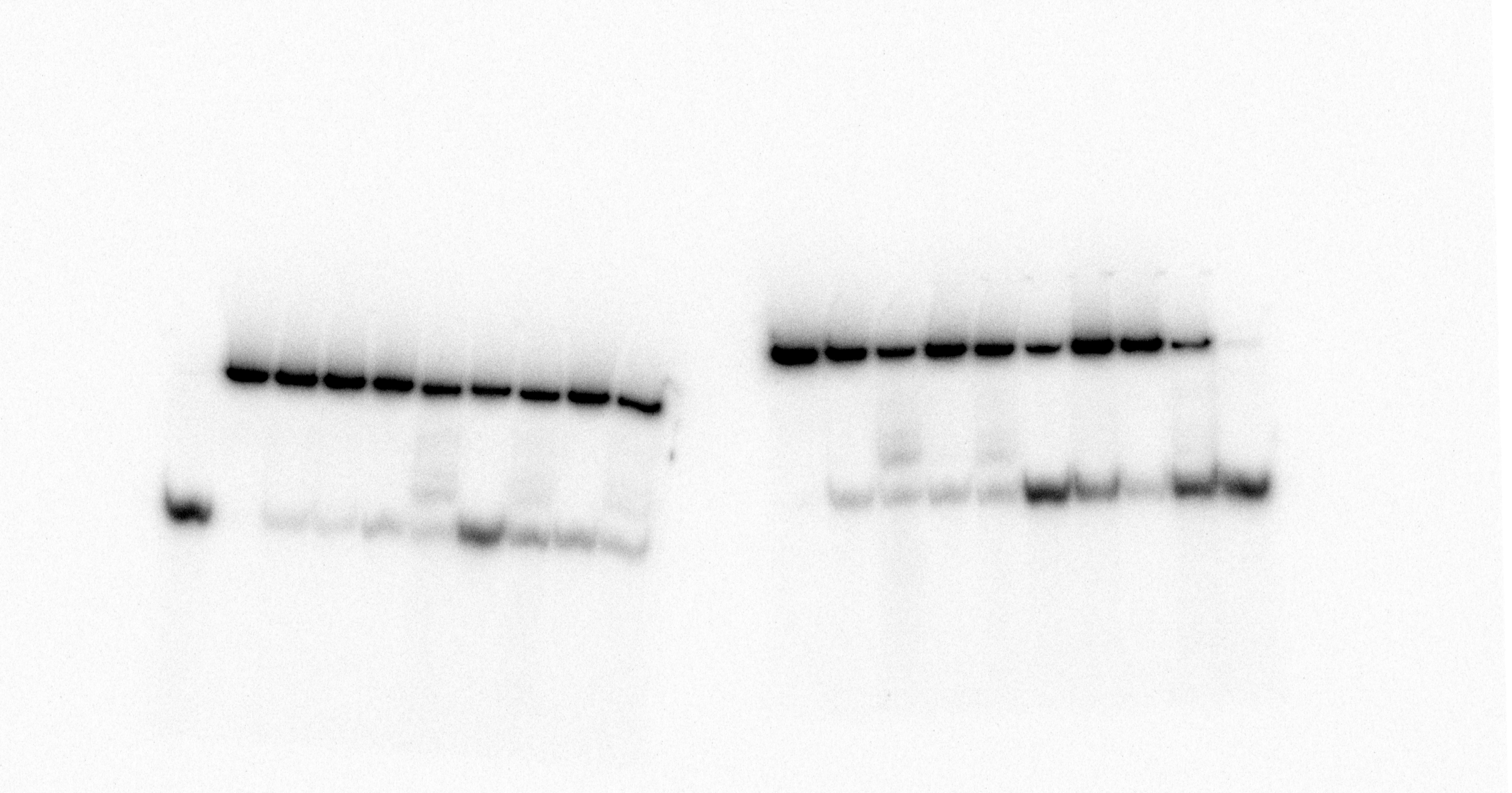

Supplement: Figure 5—source data 1. — Representative autoradiograph of the DNA unwinding reaction was conducted using a 32P-labeled ssDNA oligo annealed to the M13mp18 as DNA substrate in the presence of HROB with purified MCM8/9 or mutants as indicated. The assays were repeated at least three times. [file elife-87468-fig5-data1.zip › Figure 5 source data -1/source data 1-3.tif]

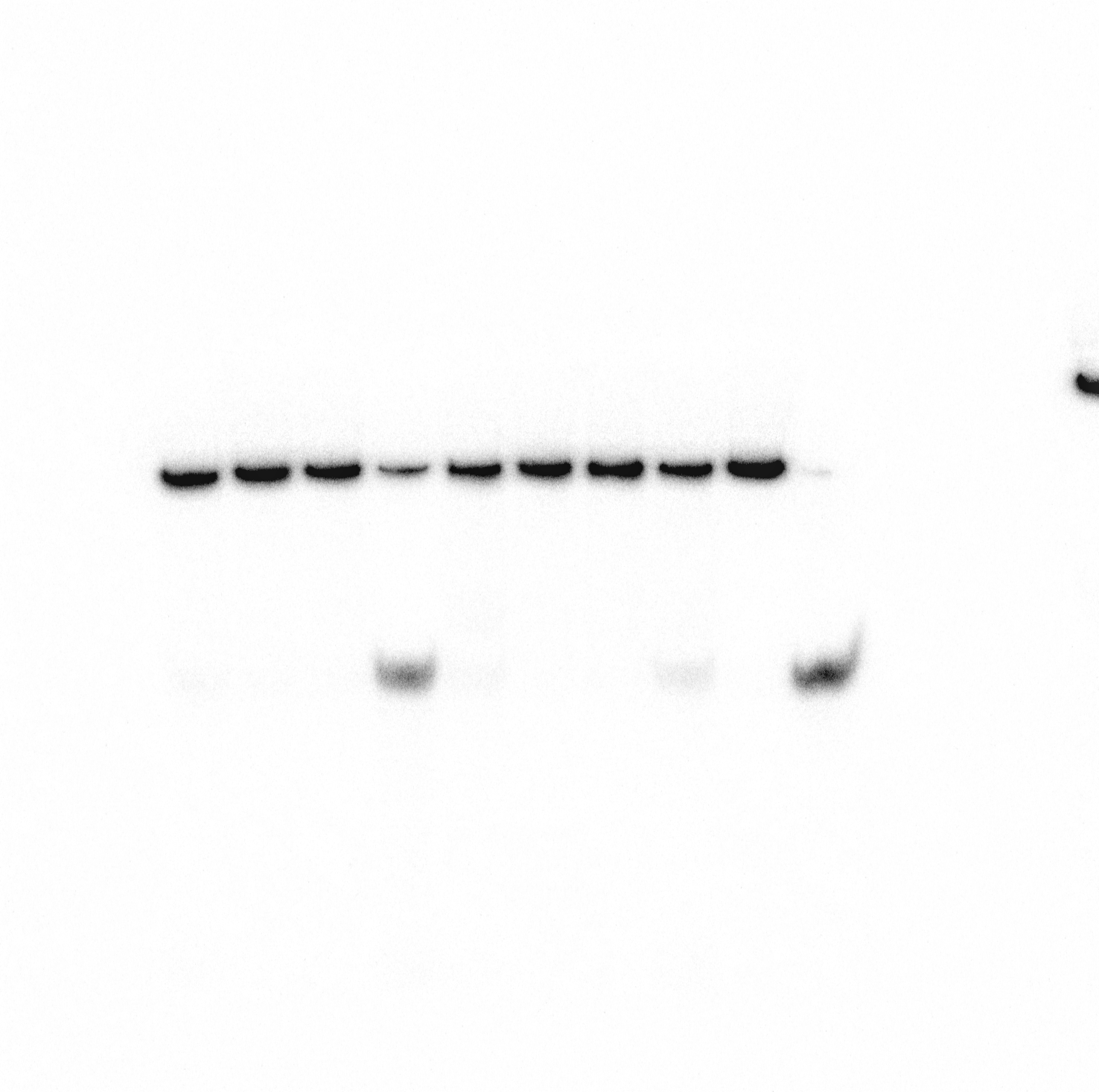

Supplement: Figure 6—source data 1. — Representative autoradiograph of the DNA unwinding reaction was conducted using a 32P-labeled ssDNA oligo annealed to the M13mp18 as DNA substrate in the presence or absence of HROB with purified MCM8/9, MCM8 N-C linker mutant (MCM8Δ369-377) or MCM9 N-C linker mutant (MCM9Δ2283-287), either alone or in combination. D-boiled DNA substrate control. The assays were repeated at least three times. [file elife-87468-fig6-data1.zip › Figure 6 source data -1/Figure 6 source data -1-1.tif]

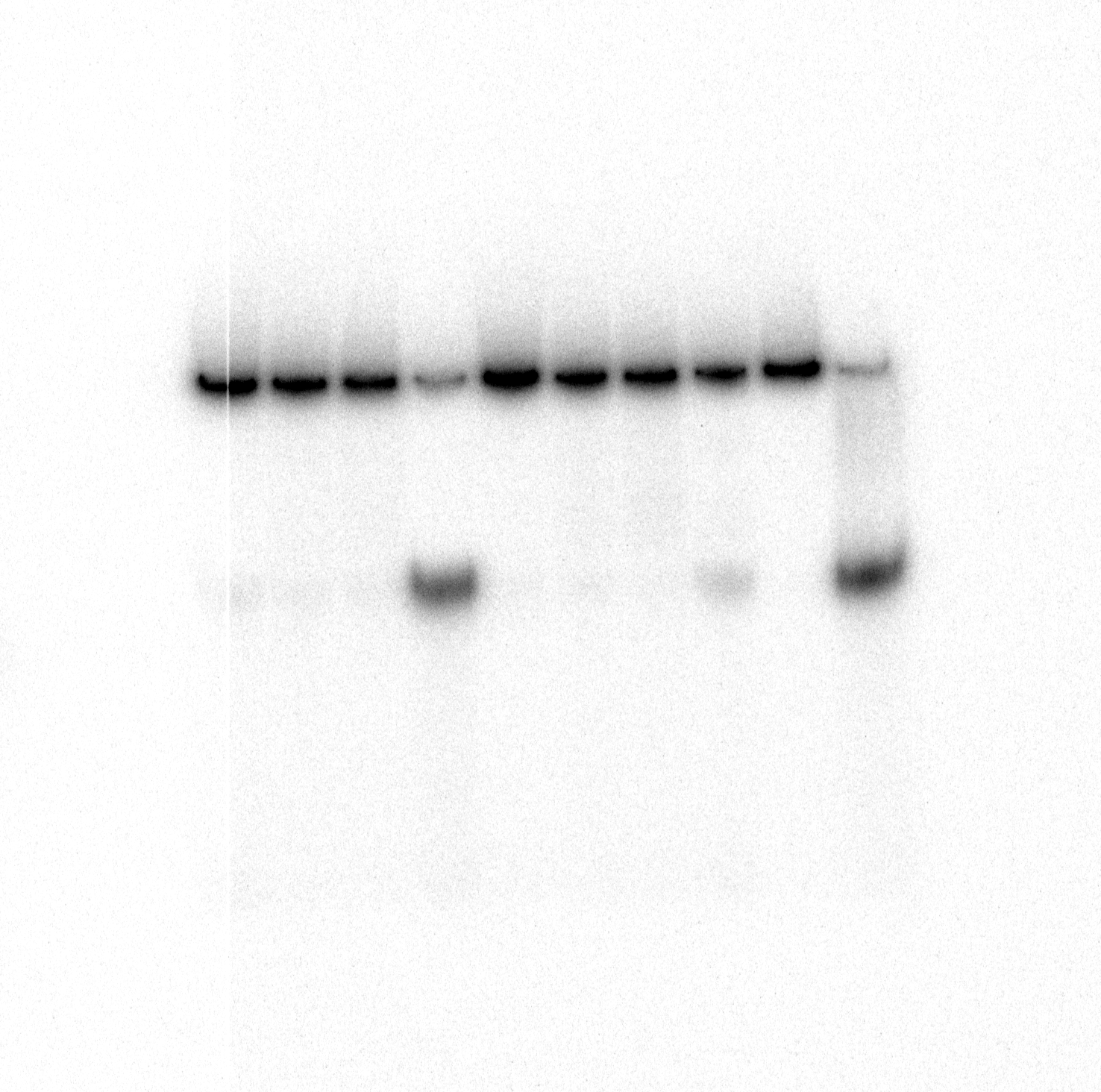

Supplement: Figure 6—source data 1. — Representative autoradiograph of the DNA unwinding reaction was conducted using a 32P-labeled ssDNA oligo annealed to the M13mp18 as DNA substrate in the presence or absence of HROB with purified MCM8/9, MCM8 N-C linker mutant (MCM8Δ369-377) or MCM9 N-C linker mutant (MCM9Δ2283-287), either alone or in combination. D-boiled DNA substrate control. The assays were repeated at least three times. [file elife-87468-fig6-data1.zip › Figure 6 source data -1/Figure 6 source data -1-2.tif]

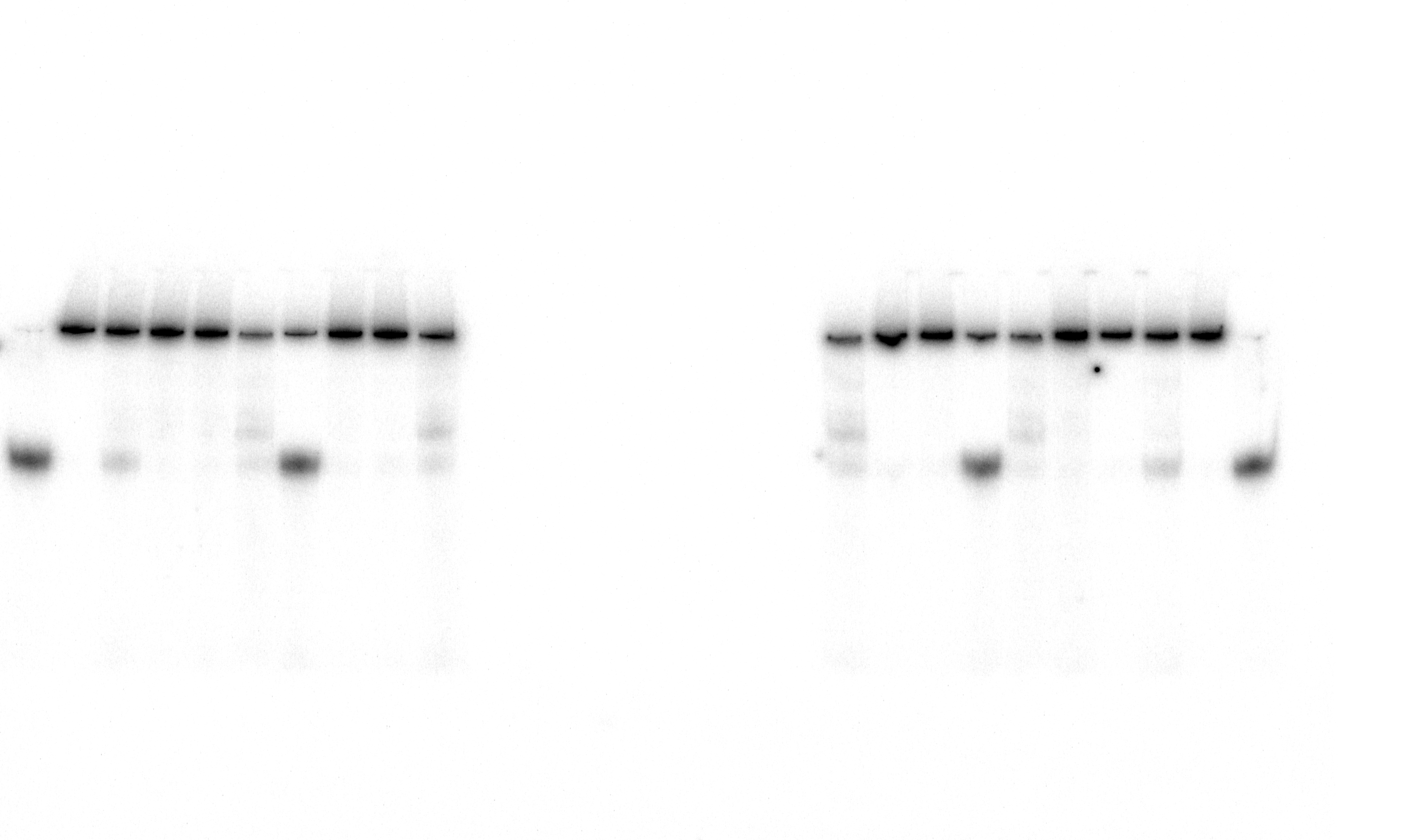

Supplement: Figure 6—source data 1. — Representative autoradiograph of the DNA unwinding reaction was conducted using a 32P-labeled ssDNA oligo annealed to the M13mp18 as DNA substrate in the presence or absence of HROB with purified MCM8/9, MCM8 N-C linker mutant (MCM8Δ369-377) or MCM9 N-C linker mutant (MCM9Δ2283-287), either alone or in combination. D-boiled DNA substrate control. The assays were repeated at least three times. [file elife-87468-fig6-data1.zip › Figure 6 source data -1/Figure 6 source data -1-3.tif]

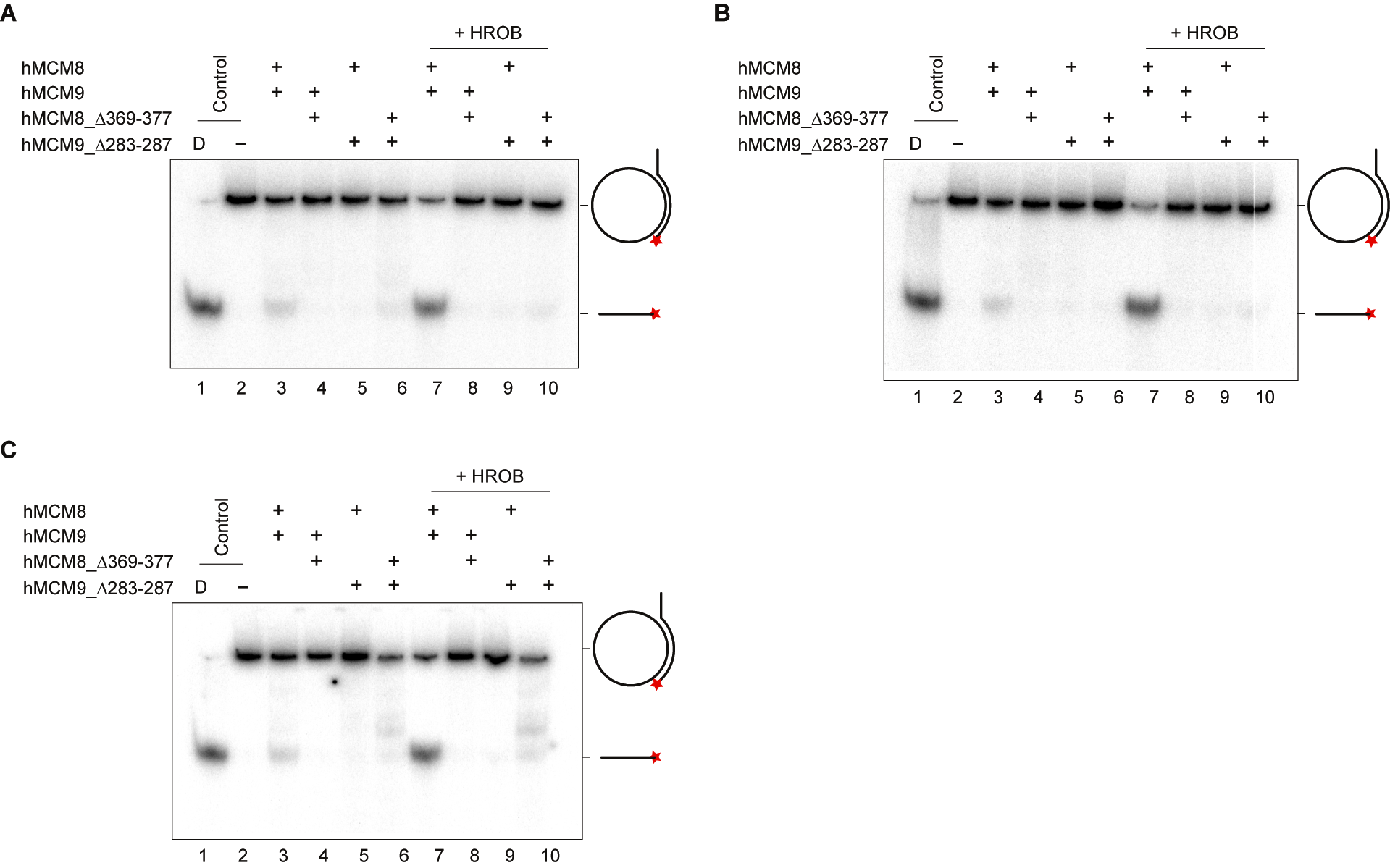

Supplement: Figure 6—source data 1. — Representative autoradiograph of the DNA unwinding reaction was conducted using a 32P-labeled ssDNA oligo annealed to the M13mp18 as DNA substrate in the presence or absence of HROB with purified MCM8/9, MCM8 N-C linker mutant (MCM8Δ369-377) or MCM9 N-C linker mutant (MCM9Δ2283-287), either alone or in combination. D-boiled DNA substrate control. The assays were repeated at least three times. [file elife-87468-fig6-data1.zip › Figure 6 source data -1/FIGURE 6 Source data 1.tif]

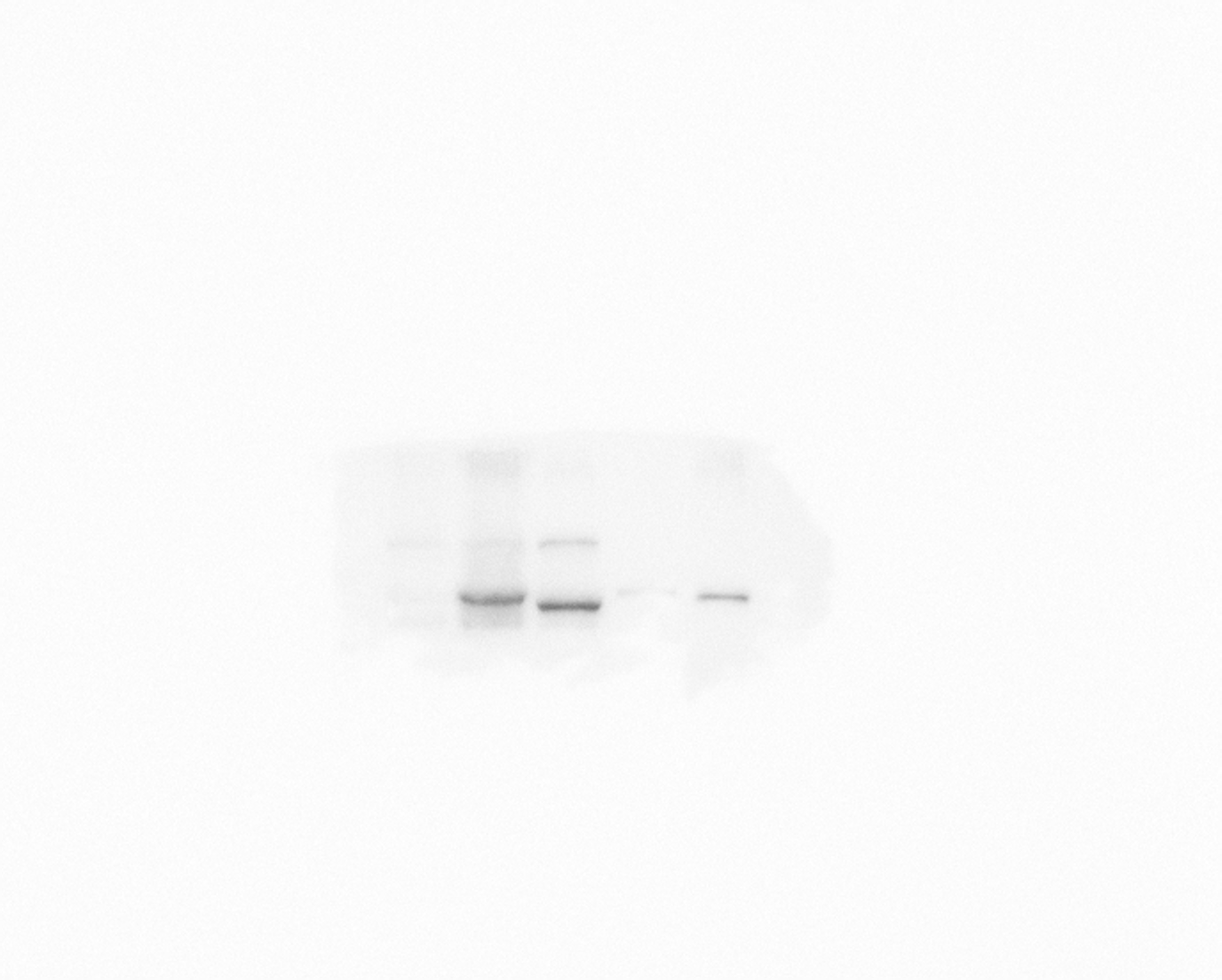

Supplement: Figure 6—source data 2. — Actin is shown as a loading control. C, control cells; Δ369–377, MCM8_Δ369–377; Δ283–287, MCM9_Δ283–287. [file elife-87468-fig6-data2.zip › Figure 6 source data -2/3s 9 89 8d9 8 8d input2.tif]

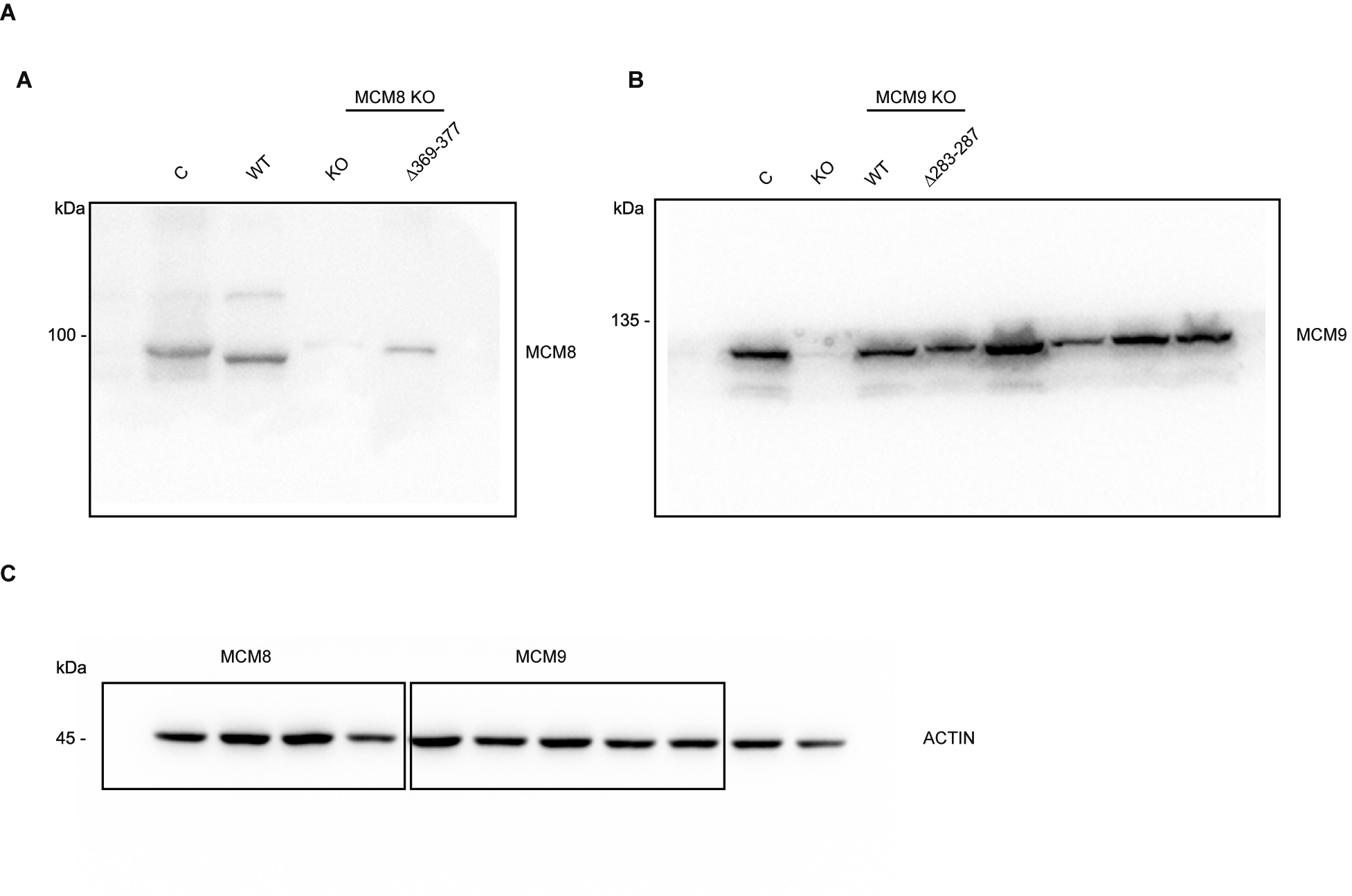

Supplement: Figure 6—source data 2. — Actin is shown as a loading control. C, control cells; Δ369–377, MCM8_Δ369–377; Δ283–287, MCM9_Δ283–287. [file elife-87468-fig6-data2.zip › Figure 6 source data -2/Figure 6 Source data 2.tif]

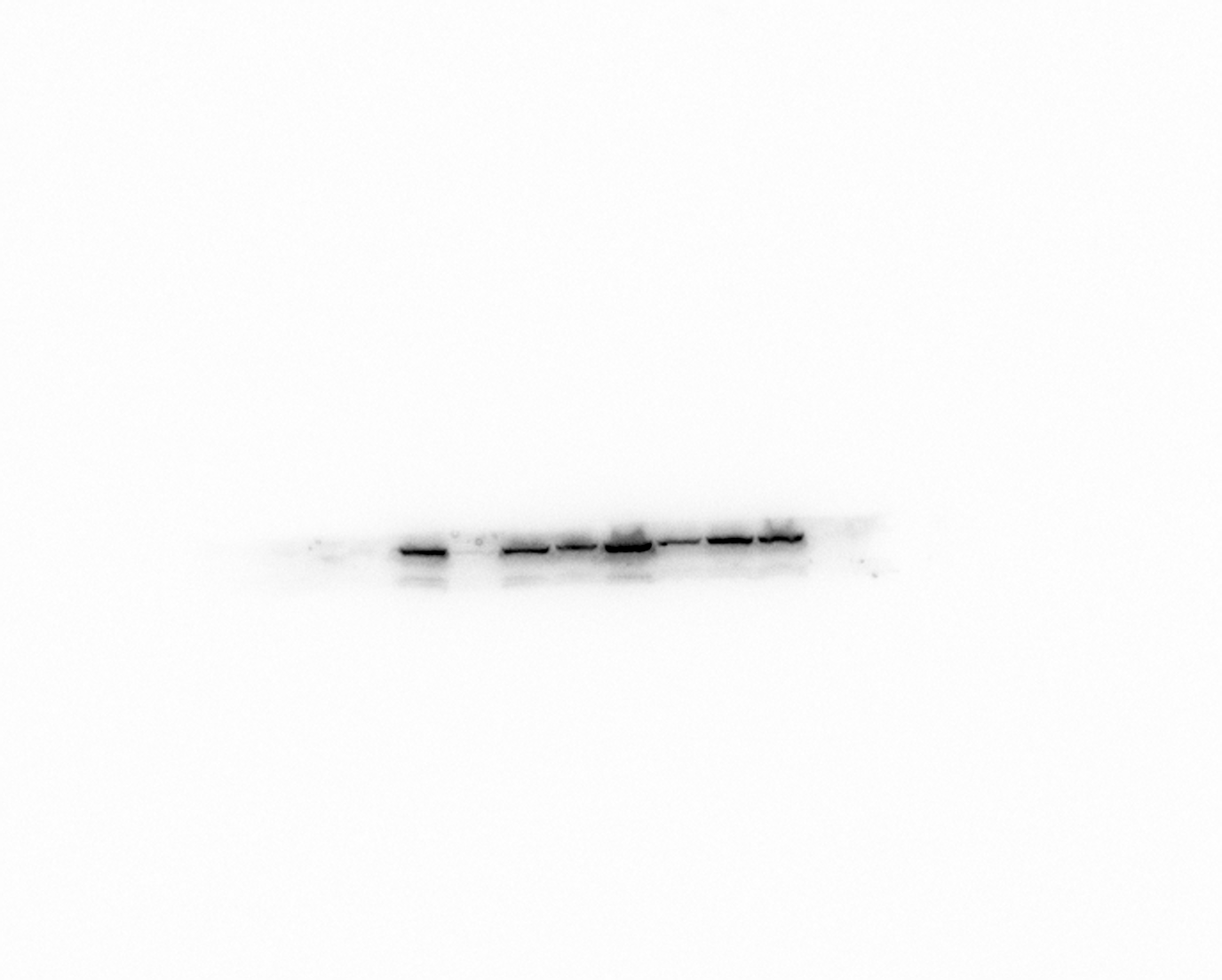

Supplement: Figure 6—source data 2. — Actin is shown as a loading control. C, control cells; Δ369–377, MCM8_Δ369–377; Δ283–287, MCM9_Δ283–287. [file elife-87468-fig6-data2.zip › Figure 6 source data -2/m8-3s.tif]

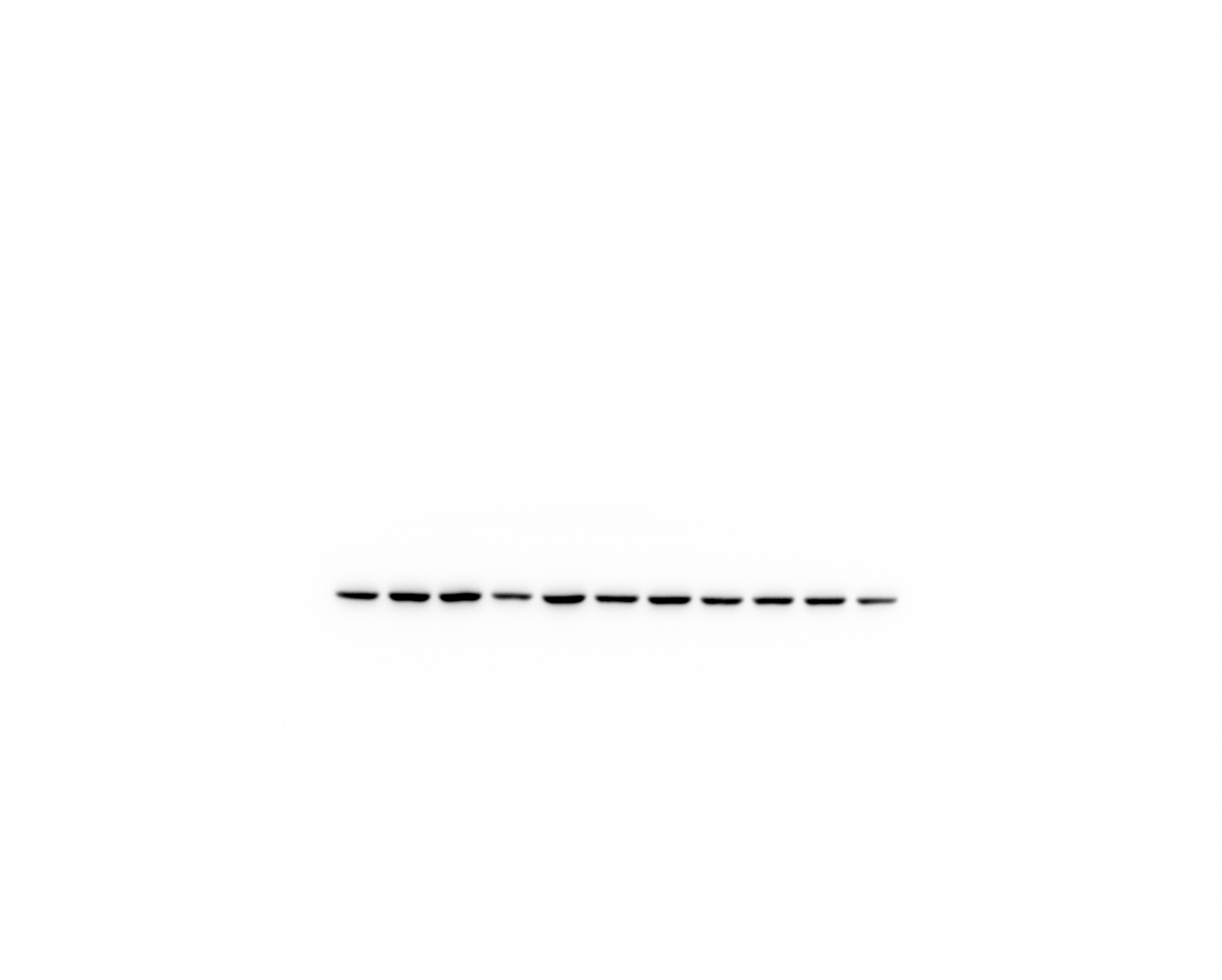

Supplement: Figure 6—source data 2. — Actin is shown as a loading control. C, control cells; Δ369–377, MCM8_Δ369–377; Δ283–287, MCM9_Δ283–287. [file elife-87468-fig6-data2.zip › Figure 6 source data -2/m9-1-10-actin.tif]
